# Translation Effectiveness of Offset Heart Rate Biofeedback as a Mindless Intervention for Alcohol Craving Among Risky Drinkers: Controlled Experiment

**DOI:** 10.2196/54438

**Published:** 2024-12-31

**Authors:** Yiran Zhao, Jatin Arora, Yujie Tao, Dave B Miller, Alexander T Adams, Tanzeem Choudhury

**Affiliations:** 1 Department of Information Science Cornell University New York, NY United States; 2 Department of Computer Science Stanford University Stanford, CA United States; 3 Department of Mechanical Engineering Tufts University Medford, MA United States; 4 School of Interactive Computing Georgia Institute of Technology Atlanta, GA United States

**Keywords:** wearable device, alcohol craving, risky drinking, digital intervention, entrainment, offset heart rate biofeedback, mindless intervention

## Abstract

**Background:**

Digital and wearable intervention systems promise to improve how people manage their behavioral health conditions by making interventions available when the user can best benefit from them. However, existing interventions are obtrusive because they require attention and motivation to engage in, limiting the effectiveness of such systems in demanding contexts, such as when the user experiences alcohol craving. Mindless interventions, developed by the human-computer interaction community, offer an opportunity to intervene unobtrusively. Offset heart rate biofeedback is an iconic type of mindless intervention powered by entrainment and can mitigate the physiological and psychological response to stressors.

**Objective:**

This work aimed to characterize the translational effectiveness of offset heart rate biofeedback on cue-elicit alcohol craving among risky drinkers.

**Methods:**

We conducted an out-of-lab, between-group, controlled experiment with 26 participants who performed harmful or hazardous drinking. The control group served as negative control and received no intervention, while the experimental group received offset heart rate biofeedback during alcohol exposure and recovery. We elicited alcohol cravings through a series of alcohol cues, including performing mental imagery, viewing alcohol images, and sniffing alcohol. We measured the physiological response to alcohol (ie, heart rate variability), self-reported craving, and self-reported anxiety. We constructed linear mixed-effects models to understand the effect of intervention during alcohol exposure and alcohol recovery after exposure. Following the linear mixed effect model, we conducted pair-wise comparisons for measures between the control and experimental groups.

**Results:**

We found that offset heart rate biofeedback significantly reduced the increase in heart rate variability (*P*=.01 and *P*=.052) and self-reported craving (*P*=.04 and *P*=.02) in response to alcohol cues. Participants’ anxiety was not affected by either the alcohol cues or the offset heart rate biofeedback.

**Conclusions:**

Offset heart rate biofeedback has the potential to immediately and unobtrusively mitigate cue-elicit alcohol craving among risky drinkers. The results of this study opened new opportunities for digital and wearable interventions to mitigate alcohol craving, either as wellness apps for risky drinkers or as digital prescriptions and integration with sensing systems for people with alcohol dependency.

## Introduction

### Background

Alcohol is the most misused substance in the United States [1]. The types of alcohol consumption behavior constitute a spectrum, from low-risk alcohol use to risky drinking and then to various levels of alcohol use disorder (AUD) [2]. Risky drinking includes binge drinking and heavy alcohol use, which in total affects more than 80 million Americans [1]. In addition, AUD affects more than 15 million Americans [1].

Traditionally, the interventions for alcohol misuse have been focused on the vigorous side of the spectrum, focusing on inpatient or outpatient rehabilitation programs [3], medication [4], and peer-led programs such as Alcoholics Anonymous [5]. Recently, cultural and lifestyle movements such as the Sober Curious Movement have increased the awareness of healthy alcohol use [6]. A large population who are on the less vigorous spectrum of unhealthy alcohol use started to be curious about treatments for alcohol use, increasing the attention on interventions for nondependent risky drinkers [7,8]. Thus, interventions that facilitate the reduction of alcohol use and the ease of craving benefit a large population.

Existing digital interventions for reducing alcohol use focused on long-term behavior change and coping. These interventions include digital psychotherapy [9-14], mindfulness practices [15,16], SMS text messages [8,17-20], and chatbots [18,21]. Despite the success of these interventions in clinical trials, they cannot provide immediate craving relief. As a result, individuals are vulnerable during acute craving episodes and are susceptible to high relapse rates [22,23]. Although the raising of mobile sensing enables just-in-time intervention systems that can detect when the users are at risk for alcohol consumption [24,25], the obtrusive nature (ie, the disruption of ongoing activities) of existing digital interventions is misaligned with the nature of at-risk contexts. As such, the potential of digital interventions and just-in-time systems can be further enabled by interventions that can achieve in-the-moment effects with minimal attention and effort.

Mindless intervention is an emerging type of intervention in the human-computer interaction community. Such intervention is delivered through mobile or wearable devices and can achieve in-the-moment effectiveness with minimal attention and engagement, hence mindless. Mindless interventions are primarily developed for regulating stress and anxiety [26-32]. The 2 main mechanisms that enable mindless interventions to regulate stress and anxiety are entrainment (eg, offset heart rate biofeedback [27,28]) and emotion regulation (eg, guided breathing [26,29-31] and affective touch [32]). Entrainment-based methods can influence the user’s physiological state by mitigating the autonomic nervous system (ANS), which is vital in regulating how the body reacts to external and internal stimuli [33].

The integration of mindless interventions into just-in-time intervention systems has the potential to mitigate cravings in-the-moment. However, careful translational evaluations are required. All existing mindless interventions were evaluated in a general population. However, the physiology of those who perform risky drinking or have AUD, regardless of severity, is different from the general, healthy population [34]. Prolonged and excessive alcohol use leads to neuroadaptations [35]. Such adaptations cause people with unhealthy alcohol use to have elevated parasympathetic nervous activity in response to alcohol cues, a phenomenon that’s not observed in the general population [34,36-40].

### Objectives

In this study, we aimed to characterize the translational effectiveness of an anxiety-mitigating mindless intervention on alcohol craving. We conducted this study as a formative step on people who performed risky drinking before experimenting with people with alcohol dependency and who needed clinical treatment. Among all mindless interventions, we chose to evaluate offset heart rate biofeedback. Offset heart rate biofeedback is delivered as a subtle vibration on the wrist [27]. Controlled laboratory evaluations have validated its ability to alter the activity of the parasympathetic branch of ANS and to mitigate anxiety [28]. We chose this intervention because (1) it best represents the merit of mindless interventions as it is nonobtrusive and requires little to no attention from the users and (2) it can be delivered by Apple Watch, allowing for scalability and low social stigma associated with use.

We hypothesized that risky drinkers who receive offset heart rate biofeedback during and after alcohol cues would experience a lower level of physiological response, a lower level of craving, and a lower level of anxiety compared with those receiving no intervention. We conducted an out-of-laboratory, controlled study with 26 participants. We found that offset heart rate biofeedback significantly mitigated both the physiological response and the subjective craving in response to alcohol cues.

### Contribution

Our study provides the first empirical evidence demonstrating that a wearable device can unobtrusively mitigate alcohol cravings in real time. This initial evidence supports the effectiveness of offset heart rate biofeedback for risky drinkers. Based on our findings, we recommend future research replicate and build on this work to translate offset heart rate biofeedback into practice as wellness apps for promoting healthy drinking habits in risky drinkers or as digital prescriptions and just-in-time interventions for preventing relapse in individuals with AUD.

## Methods

### Participants

Participants are legally drinking adults (aged 21-65 years). Participants were recruited using institution-based networks, including Slack (Slack Technologies, LLC) channels, listserves, and physical flyers, as well as social media advertisements using Meta and X. Participants were also recruited from online communities such as Facebook (Meta Platforms, Inc) groups.

All participants were screened for at-risk alcohol use by the Alcohol Use Disorders Identification Test (AUDIT) [41]. Participants were included in the study if their AUDIT score was between 8 and 14 (hazardous or harmful alcohol consumption) at the time of screening. These are the chosen thresholds because this population exhibited a similar physiological response toward alcohol cues as those who have severe alcohol dependency [36,40], which was distinctive from healthy participants [39]. We did not include participants whose AUDIT score was 15 or above to avoid unnecessary harm to individuals with more severe alcohol misuse or individuals who were under clinical treatment.

Additional inclusion criteria were individuals need to (1) be aged between 21 and 65 years, (2) reside in the United States at the time of study, and (3) be iOS (Apple, Inc) users to install the intervention system. This age group was chosen because they are the typical adult age group who can legally consume alcohol in the United States. Adolescents and the older adult population have special needs in mitigating alcohol craving, which is beyond our interests. The exclusion criteria include individuals who were (1) abstaining from alcohol at the time of the study and (2) having additional physical or mental health conditions aside from hazardous alcohol consumption. These exclusion criteria were in place to avoid exposing at-risk individuals to alcohol triggers and to minimize confounding variables to the intervention.

The study used a between-subject design. All participants underwent the identical protocol. Participants were randomly assigned to the control or experimental group at the time of study. Sex, age, and ethnicity were matched among the 2 groups (Table 1). In total, 34 participants were recruited. Furthermore, 8 participants were not included in data analysis due to interruption during the study (n=3) and low physiological data quality (n=5). As a result, 26 participants (13 in each group) were included in the data analysis. Participants’ age ranged from 21 to 52 years (mean 29.31, SD 8.65 years for control group; mean 31.69, SD 9.87 years for experimental group). Participants’ race and ethnicity included African American (experimental: n=1), Asian (control: n=6, experiment: n=3), White (control: n=7, experiment: n=6), Latinx (experiment: n=2), and multiracial between White and Asian (experimental: n=1). All participants were cisgender and binary gendered (control: 7 female individuals and 6 male individuals; experimental: 8 female individuals and 5 male individuals).

**Table 1:** Participants match the baseline characteristics of the control and experimental groups.

Variable		Control	Experimental	*P* value
**Age (years), mean (SD)**		29.31 (8.65)	31.69 (9.87)	.50
**Sex, n (%)**				.70
	Female, n (%)	7 (54)	6 (46)	
	Male, n (%)	8 (61)	5 (39)	
**Race and ethnicity, n (%)**				.30
	African American	0 (0)	1 (8)	
	Asian	6 (46)	3 (23)	
	White	7 (53)	6 (46)	
	Latinx	0 (0)	2 (15)	
	Multiracial	0 (0)	1 (6)	
**AUDIT^a^, mean (SD)**		8.85 (1.34)	9.85 (2.19)	.29
**PHQ-9^b^, mean (SD)**		5.77 (1.42)	4.85 (1.68)	.15
**GAD-7^c^, mean (SD)**		5.77 (2.68)	4.54 (1.33)	.27

^a^AUDIT: Alcohol Use Disorders Identification Test.

^b^PHQ-9: Patient Health Questionnaire.

^c^GAD-7: General Anxiety Disorder.

### Ethical Considerations

The Institutional Review Board at Cornell University approved the study in March 2020 (IRB2009009824). We obtained informed consent from the participants once the participants were screened. Participants’ data were anonymized. Participants were compensated US $25 for completing the experiment and US $5 for returning the device.

### Sample Size Determination

We conducted a priori power analysis using G*Power [42] using the effect size determined by previous studies on offset heart rate biofeedback (*d*=1.07) [27]. We balanced the minimum allowed levels for type I and type II errors by choosing 1 – β = 0.8, following the clinical, preclinical, and laboratory study guidelines [43]. The power analysis yielded the sample size requirement for 1-tailed, 2-group comparisons as n=13 and N=26.

### Study Procedure

The experiments were conducted remotely between November 2020 and August 2022. We conducted an out-of-lab, between-group, controlled evaluation. All participants performed the identical experiment protocol during the study. Participants were randomly assigned into the experimental or control group, with considerations to match age, sex, and ethnicity.

#### Screening

Once a participant signed up for the study, we conducted a 15-minute prestudy chat to introduce the study procedures and assess if the participant complied with the inclusion and exclusion criteria. We obtained informed consent from the participants if they fit the criteria and were interested in the study.

#### Device and App Distribution

The participant received a heart rate monitoring band, Polar H10 (Polar Electro Oy), through shipment or contactless drop-off. The Polar H10 was set up to collect raw electrocardiogram (ECG) signals. The participant also received instructions on how to install the experimental app onto their iPhone (Apple, Inc). If the participant owned an Apple Watch, the Apple Watch app that delivered the intervention was installed automatically with the iPhone app. Participants were asked to use laptops or desktop computers to interact with the researchers over Zoom (Zoom Communications, Inc) during the study. Researchers monitored the study procedures over Zoom.

#### Experiment

At the time of the study, researchers greeted the participants on Zoom. The researchers first introduced the study purpose with deception. To the control group participants, the researchers said that “the purpose of this study is to understand how to detect the body’s reaction to alcohol using wearable devices. We aim to collect your heart rate signal using the band we sent you.” To the experimental group participants, the researchers said that “the purpose of this study is to understand how to detect the body’s reaction to alcohol using an Apple Watch. We aim to collect your heart rate signal both using the Apple Watch and the band we sent you. You might feel a gentle tap on your wrist during the study that may or may not be at the same rate as your heart rate. It is to facilitate the collection of heart rate signal on the Apple Watch and for us to communicate and troubleshoot if the sensors are working.”

After the introduction, the researchers instructed the participants to wear Polar H10 and connect it to the intervention app. Then, the researchers sent the participants a link to the Qualtrics (Silver Lake Technology Management, LLC) survey that hosted the study contents, instructions, and surveys. Participants first filled in their prestudy questionnaires, which collected their previous week’s alcohol consumption; Patient Health Questionnaire (PHQ-9); and General Anxiety Disorder (GAD-7). Once they completed the questionnaires, participants were informed to share the browser window screen with the researchers. The researchers turned off the video and muted themselves once the participants started the study to minimize distraction to the participants.

**Figure 1:**
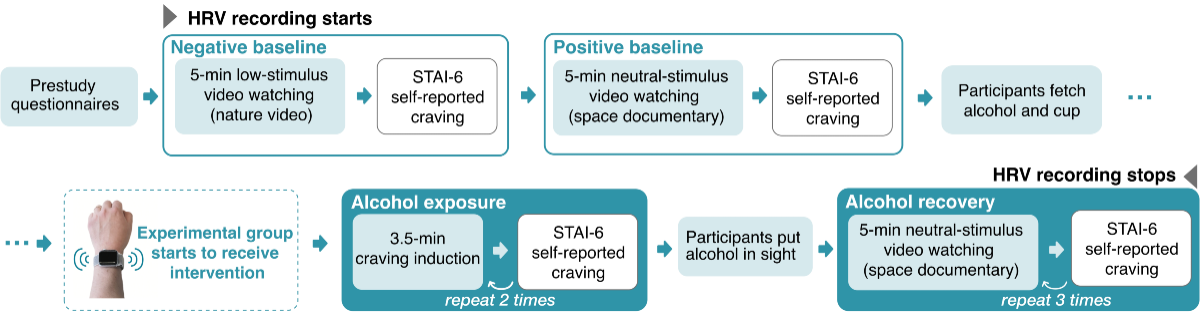
Study procedure. All participants went through the 4 sections in identical order. No alcohol was in the participants’ line of sight during the 2 baseline sections. In the 4 sections, the participants performed varied blocks of activity. HRV: heart rate variability; STAI-6: 6-item State-Trait Anxiety Inventory.

The rest of the experiment consists of four main sections (Figure 1): (1) negative baseline, (2) positive baseline, (3) alcohol exposure, and (4) alcohol recovery.

In the negative baseline section, participants performed one 5-minute block of video watching. The video was a low-stimulus nature video. The purpose of this section was to collect the participants’ responses when they were exposed to minimal stimuli.

In the positive baseline section, participants again performed one 5-minute block of video watching. The video was an excerpt from a space exploration documentary that contained affect-neutral content. The purpose of this section was to collect the participants’ responses when they were exposed to attention-getting but affect-neural stimuli.

After the positive baseline, participants were instructed to fetch the alcohol and the prepared cup. Participants in the experimental group were instructed to turn on the intervention. Deception was still in space at this time of the study. The participants were informed that the vibration on their wrists facilitated the heart rate measurements.

**Figure 2:**
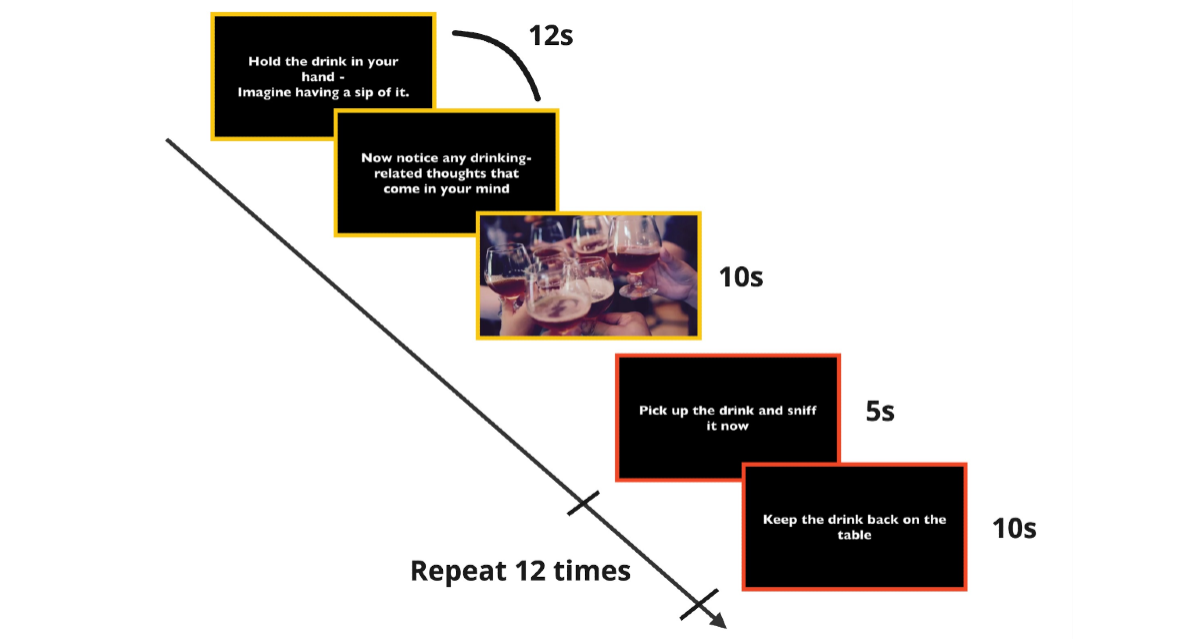
The craving manipulation included mental imagery of approximately 20 seconds as well as 5 minutes of alcohol sniffing that was repeated 12 times. One craving manipulation process lasted around 3.5 minutes.

In the alcohol exposure section, participants performed 2 identical blocks of alcohol exposure to induce craving. Each alcohol exposure block lasted 3.5 minutes (Figure 2). At the beginning of the block, participants pour a type of alcohol that they normally consume into a cup. Participants then followed an instruction video. First, to facilitate mental imagery, a white text, “Hold the drink in your hand - imagine having a sip of it,” appeared for 12 seconds on a black screen. Then, an image of wine cups and a social gathering with wine appeared for 10 seconds. Later, participants performed alcohol sniffing, instructed by a white text on a black screen: “Pick up the drink and sniff it now.” Participants continued sniffing for 5 seconds; then, the video showed “Keep the drink back on the table” as a white text on a black screen for 10 seconds to instruct the participants to stop sniffing the alcohol. Participants repeated the sniffing-putting down 12 times. The procedures (mental imagery of alcohol consumption, alcohol image, and the smell of alcohol), as well as their sequence, were well-established craving-elicit procedures adapted from previous work [40,44-46].

In the alcohol recovery section, participants put the cup with alcohol next to their computer screen so that they were no longer directly exposed to the alcohol cues but were aware of the alcohol. They performed 3 blocks of video watching. In each block, participants watched a 5-minute video excerpt from the same documentary in the positive baseline. This section aimed to collect the participants’ responses when the craving-inducing stimuli were no longer acting on them but were present in the environment. The purpose of the video watching was to use neural content to control what the participants devoted their attention to while their body and mind recovered from the alcohol exposure.

#### Debrief

After the alcohol recovery section, the researchers turned on their Zoom video and microphone and debriefed the participants. We informed the participants of the study’s true purpose: “The true purpose of the study is to evaluate an alcohol craving intervention. You might be in the control or the experimental group. If you are in the experimental group, the subtle vibration you feel on the wrist is the intervention. The vibration is 30% slower than your actual heart rate. Previous studies indicate that this kind of vibration can help down-regulate the bodily response to environmental stressors. We needed to hide the true purpose during the study because we wanted to collect natural responses to the intervention without people expecting the vibration would affect them.”

#### Deception

To control for the expectancy effect and placebo effect, we withheld the true purpose of the study and the true mechanism of the vibration from the participants. We avoided using terms such as “alcohol craving” and “intervention.” In all recruitment materials and experiment instructions, we informed participants that our study aimed to detect the bodily reaction to alcohol using wearable devices. We also said that the study was designed to simulate a scenario in which there is an opportunity to consume alcohol, but the consumption has not happened yet. We informed the participants who received the intervention that the subtle vibration is a troubleshooting mechanism to know if the sensors on the Apple Watch were working, and that the vibration may or may not be at the same rate as their heart rate [28].

### Intervention

Offset heart rate biofeedback relies on entrainment to be effective. Entrainment is the coupling and phase alignment between 2 oscillatory bodily systems [47]; it can be applied heart rate [48], brain waves [49], and movement [50]. Specifically, offset heart rate biofeedback uses a subtle vibration on the user’s wrist to entrain the user. The vibration is 30% slower or faster than the momentary heart rate [28]. In the initial controlled laboratory experiment, participants were deceived and informed that the offset heart rate represented their real heart rate [27]. In the follow-up, double-blind, controlled laboratory experiment, participants were informed that the offset heart rate feedback may or may not represent their real heart rate [28]. The results of the line of study showed that participants who received the slower heart rate feedback experienced lower self-reported anxiety and higher response in the parasympathetic branch of ANS compared with participants who received no intervention during and after stress stimulus.

We implemented the offset heart rate biofeedback on the Apple Watch following the engineering benchmark in the previous work [28]. The haptic cue is activated at a rate 30% slower than the momentary heart rate. For instance, if the individual’s heart rate is 90 beats per minute (bpm), then the Apple Watch would vibrate at 63 bpm. The upper bound of the tapping frequency is 65 bpm and the lower bound of the tapping frequency is 40 bpm [27,28]. These limits were applied to prevent the feedback from being too fast and arousal-inducing or too low such that it is natural. The tapping rate adapts to the user’s actual heart rate: if, in the previous example, the user’s heart rate drops to 72 bpm, the Apple Watch will follow along and reduce the tapping frequency to 50 bpm.

We developed a custom iOS and WatchOS app (Apple, Inc) to deliver the offset heart rate biofeedback. The app connects to an external heart rate monitor (Polar H10) through Bluetooth and streams the momentary heart rate to determine the frequency of offset heart rate biofeedback. We distributed the app through Apple’s TestFlight platform.

### Outcome Measures

We collected three types of measures: (1) heart rate variability (HRV), (2) self-reported anxiety, and (3) self-reported craving.

#### HRV Metrics

We assessed the physiological responses through HRV. HRV measures the variation between consecutive heartbeats and indicates activities in the ANS. Many HRV metrics indicate different aspects of ANS activities [51]. We used the root-mean-square of successive differences between normal heartbeats (RMSSD) and the band power of high frequency (HF; 0.15 Hz-4 Hz) because they are primary indicators of parasympathetic activity. These measures are the main indicators for alcohol response in previous studies [34,36-40].

We collected raw ECG signals using Polar H10 throughout the study. The ECG signal was processed using a pipeline established in previous work [51,52]: (1) apply a band pass filter between 0.5 Hz and 60 Hz, (2) remove baseline wander that is below 0.05 Hz, (3) detrend the signal, (4) resample the signal to 10× sampling frequency to suppress poor contact artifacts, (5) detect heart rate peaks [52], (6) remove outlying R-R interval that are less than 300 ms or greater than 2000 ms, and (7) remove ectopic heartbeats using the Malik rule [53].

We calculated a set of HRV metrics for each block using a 3.5-minute window. For blocks longer than 3.5 minutes, we used the middle 3.5 minutes. We then extracted HRV metrics from the cleaned R-R intervals for each 3.5-minute block using the *HeartPy* package [52,54].

#### Self-Reported Anxiety

We assessed self-reported anxiety with the 6-item State-Trait Anxiety Inventory (STAI-6). The original STAI measurements include 40 items, of which 20 items are used to measure state anxiety, and 20 items are used to measure trait anxiety. The 6-item short version was developed to assess state anxiety in a fast-paced environment by selecting 6 items with the strongest correlation with 20 items that measure the state anxiety [55]. We instrumented STAI-6 through Qualtrics to assess the participants’ self-reported anxiety at the end of each block.

We computed the STAI-6 score such that the 3 anxiety-present items account for positive scores (1 to 4) and the 3 anxiety-absent items account for reversed scores (4 to 1) [56]. The possible range of each participant’s STAI-6 score ranges from 6 to 24. The higher the STAI-6 score is, the higher the level of anxiety the participant experiences.

#### Self-Reported Craving

We assessed self-reported craving using a 10-item Likert scale, following the previous work’s convention [44,45]. We instrumented this self-reported craving measurement through Qualtrics at the end of each block.

### Statistical Analysis

We determined the normality of the metrics using the Shapiro-Wilk test and yielded nonnormal distributions. Thus, we treated all metrics as nonparametric variables. To assess the between-group differences in the negative baseline and positive baseline, we conducted the Mann-Whitney *U* Test.

#### Construct Linear Mixed-Effects Models

We used linear mixed-effects models (LMMs) to assess the effects of the intervention on HRV metrics (RMSSD and HF), self-reported craving, and self-reported anxiety. LMMs have been used extensively to analyze repeated-measure experiments and quantify the effect of intervention on physiological or psychological outcomes [57]. LMMs can also support the baseline adjustment for HRV variables [58]. We constructed LMMs using the lme4 package in R (R Core Team) [59,60].

We considered individual differences as the random-effect factor (ie, we used each participant as the grouping variable and fitted a random intercept for each participant). We considered the outcome measures in the negative and positive baselines, whether the participants received intervention (ie, control vs experimental), and time (ie, block 1, 2, and 3), as fixed-effect factors. We also considered that there might be interactions between intervention and time. Each time block was treated as a categorical variable following the field convention. If the interactions between intervention and time were significant, we conducted post hoc pair-wise analysis to identify if the outcome measures between the control and experimental groups differed significantly in each block. Out of the fixed effect factors, the intervention was a between-subject factor; time and baseline measures were within-subject factors.

We constructed 2 sets of LMMs—one set for alcohol exposure and one set for alcohol recovery. Analyzing them separately was critical because the exposure and recovery blocks have different stimuli and baseline outcome measures. Within the 2 sets of LMMs, one LMM was constructed for each measure (HRV RMSSD, HRV HF, self-reported craving, and self-reported anxiety). We treated self-reported anxiety and self-reported craving as continuous measurements [61]. In total, we constructed 8 LMMs, 4 to measure the effects during alcohol exposure and 4 to measure the effects during alcohol recovery.

To model the effects during alcohol exposure, the individual baselines were the outcome measures during negative baseline (no intervention, alcohol cues, or neutral information content), which were used as covariates. The reference level was the block right before the alcohol exposure (ie, the positive baseline). This way, the LMM interpreted the outcome measures in each alcohol exposure block as the change from before alcohol exposure to during alcohol exposure. We constructed the following LMM:

*Y_i_~*1 *+ Y*_0_*+ condition + time + condition : time + (*1*|participant)*

To model the effects during alcohol recovery, the individual baselines were the outcome measures during the positive baseline (no intervention or alcohol cues, but with neutral information content), which were used as covariates. This is because participants watched the excerpts from the same video during the positive baseline and the recovery blocks to minimize the effect of thoughts or mind-wandering during alcohol recovery. The reference level was the block right before the alcohol recovery (ie, the last block during alcohol exposure). The LMM considered the outcome measures in each alcohol recovery block as the change between alcohol recovery and alcohol exposure. We constructed the following LMM:

*Y_j_~*1 *+ Y*_1_*+ condition + time + condition : time + (*1*|participant)*

where *Y_j_*=measures in exposure blocks, *Y_j_*=measures in recovery blocks, *Y*_0_=measures in negative baseline (covariates), *Y*_1_=measures in positive baseline (covariates), *condition*=experimental vs control group, *time*=block number (1, 2 for alcohol exposure; 1, 2, and 3 for alcohol recovery), and (1|*participant*)=random intercept of each participant.

#### Post Hoc Pair-Wise Comparisons

After constructing the LMMs, if there were significant interactions between time and intervention, we conducted pair-wise comparisons to understand if the interaction between time and intervention was significantly different in each block. We used the *emmeans* package in R to perform these 2-tailed comparisons [62]. The pair-wise comparisons were constructed to test the following hypotheses:

1. H0: At the given time points, the experimental group participants experienced the same level of HRV RMSSD, HRV HF, self-reported craving, and self-reported anxiety as the control group participants.
2. H1: At the given time point, the experimental group participants experienced different levels of HRV RMSSD, HRV HF, self-reported craving, and self-reported anxiety than the control group participants.

## Results

### User Statistics

All participants consumed alcohol within 7 days before the study. The median number of days those participants consumed alcohol before the study was 2 days for both groups. The median number of drinks participants consumed per day 7 days before the study was 2 drinks for both groups. As shown in Table 1, there were no significant differences between the control and experimental groups in AUDIT (*P*=.29), PHQ-9 (*P*=.15), and GAD-7 (*P*=.27). During the negative baseline, the 2 groups had no significant differences in RMSSD (*P*=.68), HF (*P*=.79), self-reported craving (*P*=.42), and self-reported anxiety (*P*=.62). During the positive baseline, the 2 groups had no significant differences in RMSSD (*P*=.98), HF (*P*=.87), perceived craving (*P*=.20), and perceived anxiety (*P*=.34).

### Evaluation Outcomes

We evaluated the effectiveness of offset heart rate biofeedback on HRV, self-reported craving, and self-reported anxiety in response to alcohol cues.

#### HRV Metrics

**Figure 3:**
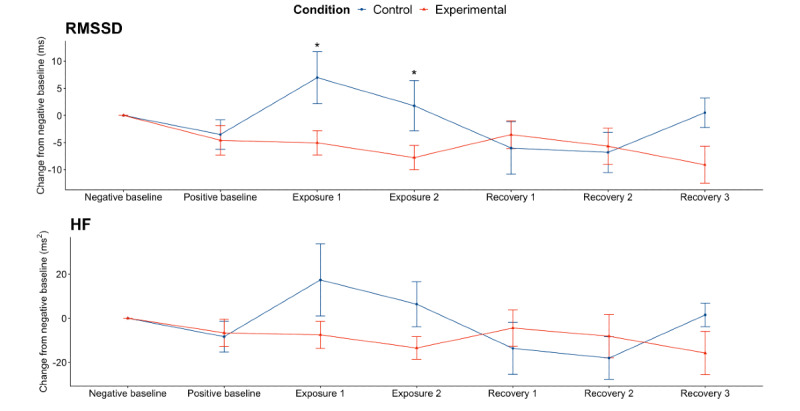
Physiological response to the alcohol cues. The results are shown as the change of root-mean-square of successive differences between normal heartbeats (RMSSD) and high frequency (HF) from each individual’s corresponding measures during the negative baseline. The points represent the mean and the error bars represent the SE. *P<.05.

We used LMMs to examine the main effects of intervention, time, and their interaction on HRV. We found a significant interaction between intervention and time on RMSSD for both blocks during alcohol exposure (Block 1: β coefficient=–10.95, 95% CI –19.48 to –2.41, *P*=.01; Block 2: β coefficient=–8.48, 95% CI –17.02 to 0.06; *P*=.052; Table 2). For HF, there was a significant interaction between intervention and time during the first block, but not the second, during alcohol exposure (Block 1: β coefficient=–26.65; 95% CI –49.80 to –3.49, *P*=.03; Block 2: *P*=.07; Table 2). Post hoc pair-wise comparisons showed that the control group experienced significantly different RMSSD (Block 1: *P*=.01; Block 2: *P*=.04; Table 3) during both exposure blocks but not for HF (Block 1: *P*=.06; Table 3). Taken together, receiving the intervention led to a lower change in RMSSD during alcohol exposures (Figure 3).

**Table 2:** Regression coefficients, 95% CI, and P values of the linear mixed-effect models estimating root-mean-square of successive differences between normal heartbeats (RMSSD), high frequency (HF), self-reported craving, and self-reported anxiety during alcohol exposure. Time was treated as a categorical variable. The corresponding outcome measures during the negative baseline were modeled as covariates. Each participant was modeled with a random intercept to account for the individual differences in baseline heart rate variability metrics, responses to alcohol cues, and responses to the intervention. The outcome measures during the positive baseline were used as reference levels. The P values indicated that the corresponding term significantly affected the outcome measures.

Variables		RMSSD	HF	Self-reported craving	Self-reported anxiety
**Random intercept**					
	β coefficient	4.84	5.21	2.72	7.28
	95% CI	−2.86 to 12.53	−13.18 to 23.60	1.45 to 3.99	3.47 to 11.08
	*P* value	.21	.57	<.001^a^	<.001^a^
**Negative baseline (covariate)**					
	β coefficient	0.76	0.73	0.30	0.43
	95% CI	0.64 to 0.91	0.55 to 0.91	0.08 to 0.53	0.06 to 0.80
	*P* value	<.001^a^	<.001^a^	.009^b^	.02^c^
**Interaction between intervention and time (Block 1)**					
	β coefficient	−10.95	−26.65	−2.12	0.46
	95% CI	−19.48 to −2.41	−49.80 to −3.49	−4.16 to −0.08	−1.75 to 2.67
	*P* value	.01^c^	.03^c^	.04^c^	.68
**Interaction between intervention and time (Block 2)**					
	β coefficient	−8.48	−21.59	−2.42	−0.46
	95% CI	−17.02 to 0.06	−44.74 to 1.57	−4.46 to −0.38	−2.67 to 1.75
	*P* value	.052^c^	.07	.02^c^	.68

^a^Significance level *P*<.001.

^b^Significance level *P*<.01.

^c^Significance level *P*<.052.

**Table 3:** P values of pair-wise comparison between the control and experimental groups in each block of the study. These comparisons were performed for root-mean-square of successive differences between normal heartbeats (RMSSD), high frequency (HF), and self-reported craving because the interactions between intervention and time were significant for these measures.

Block	*P* value		
	RMSSD	HF	Self-reported craving
Exposure block 1	.01^a^	.06	<.001^b^
Exposure block 2	.04^a^	—^c^	<.001^b^
Recovery block 1	.24	.33	.24
Recovery block 2	.45	.29	.84
Recovery block 3	—^c^	—^c^	.55

^a^Significance level *P*<.05.

^b^Significance level *P*<.001.

^c^Not applicable.

During alcohol recovery, there was a significant interaction between intervention and time on RMSSD for the first 2 blocks (Block 1: β coefficient=12.02, 95% CI 3.68-20.36, *P*=.005; Block 2: β coefficient=10.70, 95% CI 2.36-19.03, *P*=.01; Table 4), but not for Block 3 (*P*=.99). There was also a significant interaction between intervention and time on HF for the first two blocks (Block 1: β coefficient=29.05, 95% CI 7.03-51.07, *P*=.01; Block 2: β coefficient=29.74, 95% CI 7.72-51.76, *P*=.009; Table 4), but not for the third block (*P*=.82; Table 4). Post hoc pair-wise comparisons were performed on Block 1 and 2. The control and experimental group were not significantly different in Block 1 (RMSSD: *P*=.24; HF: *P*=.33; Table 3) or Block 2 (RMSSD: *P*=.45; HF: *P*=.29; Table 3). These results indicated that the intervention did not have a significant effect during alcohol recovery. Visual inspections of the trend of RMSSD and HR showed that, during alcohol recovery, both the control and the experimental group’s HRV metrics returned to the baseline levels (Figure 3), suggesting that the physiological response to the alcohol cues diminished after the cues were removed.

Overall, the results on RMSSD and HF indicated that the intervention led to significantly lower HRV metrics during alcohol exposure compared with receiving no intervention.

**Table 4:** Regression coefficients, 95% CI, and P values of the linear mixed effect models estimating root-mean-square of successive differences between normal heartbeats (RMSSD), high frequency (HF), self-reported craving, and self-reported anxiety during alcohol recovery. Time was treated as a categorical variable. The corresponding outcome measures during the positive baseline were modeled as covariates. Each participant was modeled with a random intercept to account for the individual differences in baseline heart rate variability metrics, responses to alcohol cues, and responses to intervention. The outcome measures during the last block of alcohol exposure were used as the reference level. The P values indicated that the corresponding term significantly affected the outcome measures.

Variables		RMSSD	HF	Self-reported craving	Self-reported anxiety
**Random intercept**					
	β coefficient	8.00	18.22	5.85	4.35
	95% CI	3.15 to 12.83	6.48 to 29.95	4.13 to 7.58	1.58 to 7.12
	*P* value	.001^a^	.003^a^	<.001^b^	.002^a^
**Positive baseline (covariate)**					
	β coefficient	0.92	0.92	0.46	0.61
	95% CI	0.85 to 0.99	0.83 to 1.01	0.14 to 0.77	0.40 to 0.82
	*P* value	<.001^b^	<.001^b^	.005^a^	<.001^b^
**Interaction between intervention and time (Block 1)**					
	β coefficient	12.02	29.05	1.89	0.31
	95% CI	3.68 to 20.36	7.03 to 51.07	0.23 to 3.54	−2.11 to 2.73
	*P* value	.005^a^	.01^c^	.03^c^	.80
**Interaction between intervention and time (Block 2)**					
	β coefficient	10.70	29.74	2.85	0.92
	95% CI	2.36 to 19.03	7.72 to 51.76	1.19 to 4.50	−1.50 to 3.34
	*P* value	.01^c^	.009^a^	<.001^b^	.45
**Interaction between intervention and time (Block 3)**					
	β coefficient	−0.05	2.57	2.46	1.39
	95% CI	−8.39 to 8.29	−19.45 to 24.59	0.81 to 4.12	−1.04 to 3.80
	*P* value	.99	.82	.004^a^	.26

^a^Significance level *P*<.01.

^b^Significance level *P*<.001.

^c^Significance level *P*<.05.

#### Self-Reported Craving

**Figure 4:**
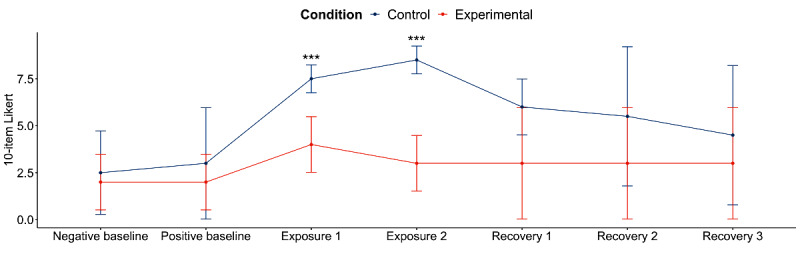
Self-reported craving in response to the alcohol cues. Results are shown as the median of the 10-item Likert scale and median absolute deviation. ***P<.001.

We used LMM to test the main effects of intervention, time, and their interactions on self-reported craving (Figure 4). During alcohol exposure, we found significant interactions between the intervention and time in both blocks of alcohol exposure (Block 1: β coefficient=–2.12, 95% CI –4.16 to –0.08, *P*=.04; Block 2: β coefficient=–2.42; 95% CI –4.46 to –0.38, *P*=.02; Table 2). Post hoc pair-wise comparisons showed that the control group and the experimental group experienced significantly different levels of self-reported craving for both blocks in alcohol exposures (Block 1: *P*<.001; Block 2: *P*<.001; Table 3). Together, these results indicated that the intervention led to significantly lower levels of self-reported craving during alcohol exposure.

During alcohol recovery, we found a significant interaction of intervention and time on self-reported craving in all 3 blocks (Block 1: β coefficient=1.89, 95% CI 0.23-3.54, *P*=.03; Block 2: β coefficient=2.85, 95% CI 1.91-4.50, *P*<.001; Block 3: β coefficient=2.46, 95% CI 0.81-4.12, *P*=.004; Table 4). Post hoc pair-wise comparisons indicated that the control and the experimental group were not significantly different for any of the 3 recovery blocks (Block 1: *P*=.24; Block 2: *P*=.84; Block 3: *P*=.55; Table 3).

Taken together, these results indicated that the intervention led to significantly lower levels of self-reported craving during alcohol exposure. During alcohol recovery, both groups returned to baseline levels of self-reported craving as the alcohol cues were removed.

#### Self-Reported Anxiety

**Figure 5:**
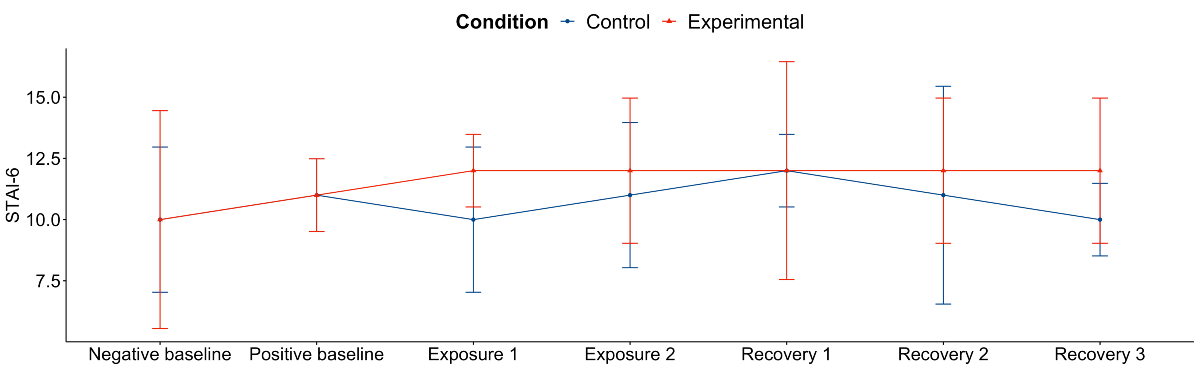
Self-reported anxiety in response to the alcohol cues. Results are shown as the median 6-item State-Trait Anxiety Inventory score (6-24) and median absolute deviation. STAI-6: 6-item State-Trait Anxiety Inventory.

We used LMMs to understand the main effects of intervention, time, and their interactions on self-reported anxiety. During alcohol exposure, there was no significant interaction between intervention and time (Block 1: *P*=.68; Block 2: *P*=.68; Table 2). During alcohol recovery, there was no significant interaction between intervention and time (Block 1: *P*=.80; Block 2: *P*=.45; Block 3: *P*=.26; Table 4). Overall, the LMMs indicated that neither intervention nor time had a significant effect on anxiety. A visual inspection of the median and absolute deviation on anxiety also showed that anxiety had little change over the different segments of the study (Figure 5).

## Discussion

### Principal Findings

This paper evaluated the translational effectiveness of offset heart rate biofeedback (“the intervention”), a mindless intervention that had shown effect in mitigating anxiety, as an alcohol craving intervention. The results indicated that the intervention significantly lowered the physiological response to alcohol cues and reduced the self-reported craving during alcohol exposure among individuals who performed harmful and hazardous drinking (ie, risky drinkers).

The results from our study exhibited 3 main trends. First, participants who received the intervention experienced a steady level of parasympathetic activity (ie, HRV RMSSD and HRV HF) during alcohol exposure. In contrast, the participants who did not receive the intervention experienced elevated parasympathetic activity during alcohol exposure. Second, the self-reported craving followed the same trend as the physiological response. Third, the self-reported anxiety did not follow the same trend as the physiological response or the self-reported craving; neither alcohol exposure nor the intervention had a significant impact on self-reported anxiety.

The observations on the physiological response and self-reported craving are in alignment with the findings in previous studies—individuals who were moderately or heavily dependent on alcohol experienced elevated parasympathetic activity in response to alcohol cues; such elevation was higher than healthy drinkers [34,36-40]. A higher increase in cue-elicit parasympathetic activity is associated with a higher level of craving [37].

The trend in self-reported anxiety was not in alignment with previous studies. In the previous work that implemented offset heart rate biofeedback as an anxiety intervention [27,28,30], the intervention led to increased parasympathetic activity and lower self-reported anxiety. In contrast, the intervention implemented in our study led to a stable parasympathetic activity while having no effect on self-reported anxiety. This observation, although not aligned with previous studies, was sensible because this study did not manipulate alcohol craving through anxiety. It is possible that alcohol craving would lead to elevated anxiety [63]. However, such response was not observed in this population, possibly because their dependency on alcohol was moderate.

Based on the 3 observed trends, we hypothesized that the intervention did not mitigate craving by reducing the participants’ anxiety but by stabilizing the physiological response elicited by alcohol cues. The reduced change in parasympathetic activity could subsequently lead to a stabilized subjective experience of craving (as denoted by self-reported craving). Following this hypothesis, the previously observed effect on stress and anxiety could be that the entrainment signal stabilizes the stress response (ie, sympathetic activation in response to stress manipulation), as shown in higher parasympathetic activity in those who received the intervention [28] during stress manipulation. The little increase in self-reported anxiety levels during and after stress manipulation could be an effect of the stabilized physiological response, similar to what was observed in this study [27,28]. To verify this speculation on the mechanism behind offset heart rate biofeedback, future studies should replicate the study procedure with anxiety-elicited craving and compare the physiological effect with this study.

As a formative study, this study aimed to characterize the effect of offset heart rate biofeedback on cue-elicit alcohol craving in a controlled setting. The promising trends in reducing both physiological and self-reported cravings suggest new possibilities for digital and wearable interventions targeting alcohol misuse. Unlike existing digital interventions, offset heart rate biofeedback has the potential to directly reduce craving unobtrusively. Compared with digital psychotherapy, push messages, or chatbots, this intervention provides a more direct and immediate influence on craving. Compared with mindfulness exercises, this intervention requires much less engagement and attention from the user. As such, offset heart rate biofeedback is particularly impactful in day-to-day living and outpatient settings. For risky drinkers, offset heart rate biofeedback can be integrated into wellness apps to help control the urge to drink and promote healthier alcohol use. For individuals with AUD, offset heart rate biofeedback can serve as a digital prescription in outpatient settings, either as a standalone tool for immediate craving relief or integrated into just-in-time sensing systems to prevent relapse during high-risk situations.

### Limitations and Future Work

This study raised new questions for future research to validate its generalizability and further investigate the mechanism of offset heart rate biofeedback. First, for risky drinkers, the results of this study served as the initial evidence of offset heart rate biofeedback in craving mitigation. Future studies should replicate its results using alternative cue-elicitation methods and test its effectiveness in naturalistic settings. Second, this study deliberately evaluated participants with risky drinking behaviors (ie, those who were not under the clinical definition of AUD). This limits the generalizability of the results in this study to the clinical population. Thus, future studies should replicate this study in the clinical population to understand whether this intervention is effective among individuals with AUD. Finally, replicating this study with anxiety-elicited craving can deepen the understanding of the mechanisms behind offset heart rate biofeedback. A more profound theoretical framework will bolster confidence in evaluating this intervention in a population with severe alcohol dependency.

### Conclusions

This paper presents the translational evaluation of offset heart rate biofeedback, an iconic type of mindless intervention, extending its application from anxiety mitigation to alcohol craving mitigation. Through an out-of-laboratory, controlled, between-group evaluation with participants who practiced harmful or hazardous drinking, we discovered that offset heart rate biofeedback significantly reduced both physiological responses to alcohol cues and self-reported cravings during alcohol exposure. This paper marks the first empirical evidence that a wearable device can unobtrusively and immediately mitigate alcohol cravings, creating new opportunities for digital interventions to support a broad range of individuals—from risky drinkers to those with AUD. Following this formative work, future studies should investigate the generalizability of this study’s results by conducting evaluations in more naturalistic settings and among populations with more severe alcohol misuse.

## Abbreviations

ANS: autonomic nervous system
AUD: alcohol use disorder
AUDIT: Alcohol Use Disorders Identification Test
bpm: beats per minute
ECG: electrocardiogram
GAD-7: General Anxiety Disorder
HF: high frequency
HRV: heart rate variability
LMM: linear mixed-effects model
PHQ-9: Patient Health Questionnaire
RMSSD: root-mean-square of successive differences between normal heartbeats
STAI-6: 6-item State-Trait Anxiety Inventory

## Data Availability

The datasets generated and analyzed during this study are available from the corresponding author on request.
